# Gut Sphingolipid Composition as a Prelude to Necrotizing Enterocolitis

**DOI:** 10.1038/s41598-018-28862-4

**Published:** 2018-07-20

**Authors:** B. Rusconi, X. Jiang, R. Sidhu, D. S. Ory, B. B. Warner, P. I. Tarr

**Affiliations:** 1 Department of Pediatrics, Washington University in St. Louis School of Medicine, St. Louis, MO USA; 2 Department of Medicine, Washington University in St. Louis School of Medicine, St. Louis, MO USA; 3 Department of Molecular Microbiology, Washington University in St. Louis School of Medicine, St. Louis, MO USA

## Abstract

Necrotizing enterocolitis (NEC) remains a major challenge in neonatology. Little is known about NEC pathophysiology apart from the presence of pre-event gut dysbiosis. Here, we applied broad range metabolomics to stools obtained 1–5 days before NEC developed from 9 cases (9 samples) and 19 (32 samples) controls matched for gestational age at birth and birth weight. The 764 identified metabolites identified six pathways that differ between cases and controls. We pursued sphingolipid metabolism because cases had decreased ceramides and increased sphingomyelins compared to controls, and because of the relevance of sphingolipids to human inflammatory disorders. Targeted analysis of samples from 23 cases and 46 controls confirmed the initial broad range observations. While metabolites provided only 73% accuracy of classification by machine learning, hierarchical clustering defined a sphingolipid associated grouping that contained 60% of the cases but only 13% of the controls, possibly identifying a pathophysiologically distinct subset of NEC. The clustering did not associate with any of the analyzed clinical and sample variables. We conclude that there are significant changes in sphingolipid metabolism components in pre-NEC stools compared to controls, but our data urge circumspection before using sphingolipids as broadly applicable predictive biomarkers.

## Introduction

Necrotizing enterocolitis (NEC) is a devastating necroinflammatory injury of the intestines that affects very low birthweight (VLBW) infants. Its 2–7% incidence in high-income countries, treatment (i.e., massive bowel resection for severe cases), and 22–38% case fatality rates have changed little in four decades^1,2^. Indeed, NEC is now the chief cause of death in VLBW infants who survive the first 14 days of life^3^. Furthermore, NEC survivors experience higher rates of functional impairment throughout childhood^4^. Gestational age at birth, antibiotic treatment in the first week of life, and lack of human milk feeding remain the only factors that are consistently associated with this event^5–7^. Prophylactic measures have focused on encouraging use of human milk, probiotics, truncating antibiotic use in the first week of life, and holding feeds during transfusions. However, the value of these strategies remains strongly debated, recommendations are under continuous development, and there is considerable inter-center variability in implementation^3,5,8–16^.

Efforts to identify a pre-NEC bacterial signature converge on overabundance of Gram-negative bacilli and relative paucity of obligate anaerobic bacteria^17–24^. This microbial contribution to the development of NEC opens new avenues for early intervention, but in view of day-to-day variations in microbial content^25^, it is difficult to rely solely on this measurement in a single specimen as being determinative of risk. Furthermore, microbial content does not illuminate host physiology prior to NEC onset. In an effort to elucidate the pathophysiology of NEC and possibly identify metabolic markers of at-risk infants, we initiated a broad range metabolomics study, followed by targeted metabolomics to confirm the initially identified molecules of interest.

## Results

### Patient population and clinical variables

Overall, we analyzed samples from 24 infants with Bell’s Stage ≥II NEC, 5 infants Bell’s Stage I NEC, and 67 controls who were matched for gestational age at birth, birth weight, and the day-of-life that samples were produced (see Supplementary Fig. S1). Demographic and clinical data for the targeted metabolomics specimen set (Table 1) reflect previous reports for this cohort^22,25,26^. A set of implicated risk factors for NEC, such as transfusions^27^, feeding^5,28^, inotrope^29^, and antibiotic^11^ use were included in our analysis to account for possible confounding factors.

**Table 1:** Variables Included in HAllA Analysis.

	Cases (23)	Controls (46)	Unit or Classifier
*Infant variables*			
Cluster	14 (60.9%)	6 (13%)	SLA-cluster
DOL	24 (18.5–47.5)	23 (17.25–32)	days
BW	800 (720–955)	840 (662.5–927.5)	grams
GA	25.9 (24.7–27.35)	25.5 (25–27.5)	weeks
Gender	9 (39.1%)	15 (32.6%)	female
Multiple birth	4 (17.4%)	8 (17.4%)	
Delivery	4 (17.4%)	17 (37%)	vaginal
*NEC variables*			
Sampling Interval*	3 (13%)/4 (17.4%)	7 (15.2%)/6 (13%)	2,3 days
Stage	6 (26.1%)/17 (73.9%)		II, III
Surgical	13 (56.5%)		
Outcome	8 (34.8%)	1 (2.2%)	death, discharge
*Medication variables*			
Antibiotic exposure %	42.3 (35.6–50.1)	45 (22–64)	% of days p.s.
Antibiotics	2 (8.7%)	12 (26.1%)	a.s.
Interval Antibiotics %	0 (0-0)	0 (0–53)	7 days p.s.
Transfusion	2 (8.7%)	0	a.s.
Transfusion Volume	46 (18–59)	24 (0–39.8)	mL
Transfusion Interval	11 (2.5–15.5)	7.5 (3–18)	Days p.s.
Total transfusions	4 (1–6)	2 (0–3)	Transfusion events
Iron	3 (13%)	13 (28.3%)	a.s.
Iron %	0 (0-0)	0 (0–8.8)	% of days p.s.
Iron Interval	0 (0-0)	0 (0-0)	d.s.
Inotropes	3 (13%)	5 (10.9%)	a.s.
Inotropes %	0 (0–24)	0 (0–7.4)	% of days p.s.
Inotrope Interval	11 (1.5–16)	7 (0–14.8)	d.s.
*Feeding variables*			
Any HM	15 (65.2%)	31 (67.4%)	a.s.
HM & Formula	4 (17.4%)	3 (6.5%)	a.s.
HM %	100 (67–100)	100 (89–100)	% of days p.s.
Fortifier	16 (69.6%)	30 (65.2%)	a.s.
Fortifier %	32 (7.5–63)	67 (0–87)	% of days p.s.
HM Interval	15 (65.2%)	29 (63%)	2 days p.s.
HM & Formula Interval	5 (21.7%)	7 (15.2%)	2 days p.s.
Fortifier Interval	8 (34.8%)	29 (63%)	2 days p.s.
Fortifier mix Interval	8 (34.8%)	2 (4.3%)	2 days p.s.

Data are median (IQR) or sample size (%). *Days between sampling and NEC. p.s.: prior to sampling, a.s.: at sampling, d.s.: days since last treatment prior to sampling, HM: Human milk is from any source.

### Broad Range Metabolomics of Pre-NEC Stool Samples and Controls

**Figure 1:**
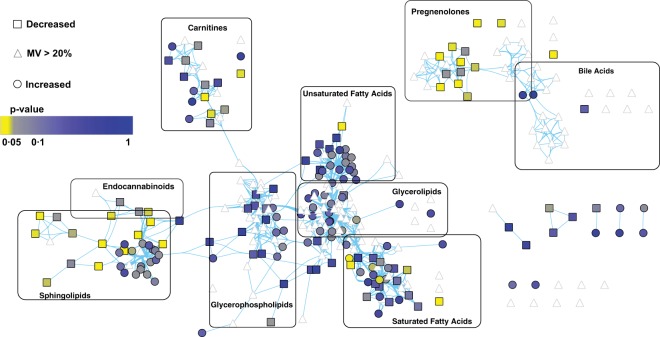
Network analysis of fecal lipid metabolites from NEC cases and controls. Broad range assay metabolites detected and clustered based on biochemical (KEGG) and chemical similarity (PubChem) connections (blue lines) denote a high density of significant changes (p < 0.05) in members of the sphingolipid and pregnenolone pathways Lipids that significantly changed between cases (1–5 days prior to NEC onset) and controls are in yellow (p < 0.05) and grey (p < 0.1). Statistical significance was determined by two-sided Welch’s test with Benjamini Hochberg (BH) correction for multiple comparison. Grey triangles represent lipids with >20% missing values, squares represent lipids decreased in cases, and circles represent lipids that are increased. Lipids with limited or no network connection are depicted as individual points in the right lower quadrant of the figure.

For the broad range analysis, we chose stool specimens closest to NEC onset (but not from the day of NEC), but no more than five days preceding NEC onset, from nine cases, and 32 specimens from 19 controls that were matched for day of life of production of these specimens (see Supplementary Fig. S1 and Supplementary Table S1). UPLC-MS/MS identified over 700 metabolites (see Supplementary Table S2), consisting of lipids (n = 312), amino acids (n = 180), carbohydrates (n = 50), xenobiotics (n = 91) and other metabolic pathways in these 41 stools. However, only 419 metabolites were consistently (i.e., less than 20% missing values) present in all samples and advanced to subsequent analysis (see Supplementary Table S2, yellow). We then performed network analysis based on the corresponding KEGG biochemical pathway identifier and chemical similarity to visualize changes. A univariate analysis with false discovery rate correction of the data highlighted a limited set of pathways that differed significantly between the nine case stools and the 32 control stools (Fig. 1). Members of the pregnenolone, carnitine, and sphingolipid pathways were over-represented among significant hits (p < 0.05), suggesting nonrandom metabolic alterations rather than random noise. Members of the sphingolipid pathway were of particular interest, as they demonstrated a metabolic shift towards increased sphingomyelins and decreased ceramides. These changes could arise from either decreased degradation or increased production of sphingomyelins. A secondary analysis of the broad range metabolic data by a modified principal component analysis (PCA) mainly identified lipid pathways that were enriched in significant hits, even after a more stringent Bonferroni correction (see Supplementary Fig. S2). When significant differences in metabolite presence were identified, the cases more commonly demonstrated decreased relative abundance of molecules of interest.

NEC has been linked to microbial dysbiosis and intestinal inflammation^22,24,30–33^, and sphingolipids modulate membrane barrier function^34^ and immune cell trafficking^35–39^. However, how the altered microbial community is established and how the variant community interacts with the immune system remain unclear. Although bacterial sphingolipids have been implicated in other gastrointestinal pathologies, they appear to have distinctly different profiles and functions from sphingolipids of human origin^40^. Therefore our broad range data prompted us to apply a targeted approach to sphingolipids, based on the initial findings from the broad range analysis, and the likelihood that the pathways identified were of host origin.

### Targeted Sphingolipids Metabolomics for Classification

**Figure 2:**
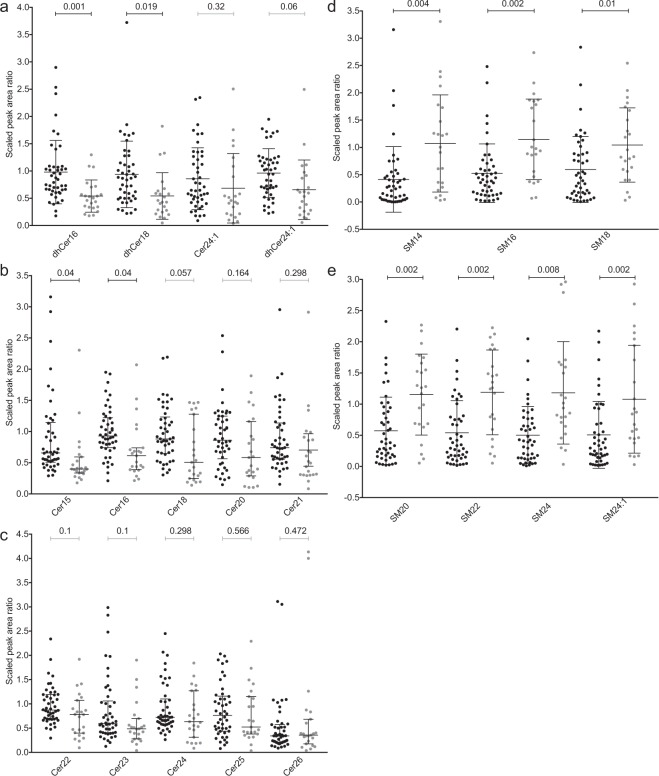
Targeted sphingolipid detection in pre-event stool. (**a**–**c**) scaled peak area ratios of ceramides to internal standard ceramide (Cer17:0). (**d,e**) scaled peak area ratios of sphingomyelins to deuterated internal standard sphingomyelins (SM18:1-d9). Scatterplot of scaled values with median and interquartile range were compared by two-sided Welch’s t-test with BH multiple test correction (p < 0.05). Black circles represent controls and grey circles represent cases.

We used targeted metabolomics to detect 14 different ceramides (Cer) and seven different sphingomyelins (SM) in pre-event stools (1–3 days) in our expanded analysis of 23 cases and 46 matched controls. Because the broad range analysis was limited by repeat sampling in the controls, we included only single time points for each control in the targeted assay and expanded the control population. As observed in the broad range metabolic approach, ceramides were significantly lower and sphingomyelins significantly higher, in case specimens (Fig. 2). However, the expanded numbers of controls (N = 46) did indeed result in a wider range of values. We next considered the possibility that in the enlarged control specimen set, we inadvertently included infants intolerant of feeds or who even had Stage I NEC, even though they were not designated as such. In the event that Stage I NEC cases were unintentionally included, specimens from such subjects might have contributed to the lesser effect, if Stage I NEC is on a pathophysiologic continuum that progresses to overt NEC. Note that Stage I NEC cases were intentionally excluded from our initial analyses and prior publications, as there is no consensus case definition for this entity^41^. However, targeted metabolomics analysis on samples from five infants who soon thereafter developed Stage I NEC and corresponding controls, as designated in their medical records, offered no evidence that the control set might have been influenced by these minimally clinically affected Stage I NEC cases (see Supplementary Fig. S3). In other words, the stools from infants soon-to-be assigned to the category of Stage I NEC had values that fell within the range observed in stools from the controls, but not the cases (see Supplementary Fig. S3). This suggests that only Stages II and III NEC are associated with changes in sphingomyelins and ceramides, consistent with emerging thinking that feeding intolerance and Stage I NEC fundamentally differ from what is currently defined as Stage II and III NEC^41,42^.

We next asked if the altered sphingolipid profiles could assist machine-learning classification of NEC risk. The algorithms were trained on a dataset with known classification (NEC ≥ Stage 2 vs. control). If good specificity and sensitivity are achieved, then the model can be validated with a second cohort. We applied seven machine learning algorithms encompassing diverse approaches. Over the 10 repeated training sessions the k-nearest neighbor (KNN) model had the greatest accuracy (73%) based on the 21 sphingolipids (Table 2). However, the resulting greater standard deviation and false positive rates (see Supplementary Table S3) generated in cross-validation lead us to conclude that the model based on sphingolipids does not currently offer sufficient discriminatory power to identify all cases of NEC, an attribute that would be needed for further development of biomarkers.

**Table 2:** Ranking of Machine Learning Models for NEC Classification.

Rank							
Iteration	1	2	3	4	5	6	7
1	JRip	KNN	PLS	J48	NB	RF	SVMradial
2	KNN	JRip	NB	PLS	RF	J48	SVMradial
3	KNN	PLS	JRip	J48	NB	RF	SVMradial
4	KNN	PLS	JRip	NB	J48	RF	SVMradial
5	KNN	PLS	J48	JRip	NB	RF	SVMradial
6	KNN	J48	JRip	PLS	RF	NB	SVMradial
7	KNN	PLS	NB	JRip	J48	RF	SVMradial
8	KNN	PLS	JRip	NB	J48	RF	SVMradial
9	KNN	JRip	PLS	NB	J48	RF	SVMradial
10	KNN	PLS	JRip	J48	NB	RF	SVMradial
Total	KNN	PLS	JRip	NB	J48	RF	SVMradial
Average Accuracy	75 ± 15	72 ± 16	71 ± 17	70 ± 17	69 ± 17	68 ± 17	57 ± 19
Average ROC	77 ± 21	77 ± 23	70 ± 18	75 ± 22	71 ± 20	78 ± 18	65 ± 25

KNN: k-nearest neighbor, PLS: partial least squares, RF: random forest, NB: naïve Bayes, SVMradial: support vector machine radial, J48: C4.5-like decision algorithm, JRip: repeated incremental pruning algorithm, ROC: receiver operating characteristic.

**Figure 3:**
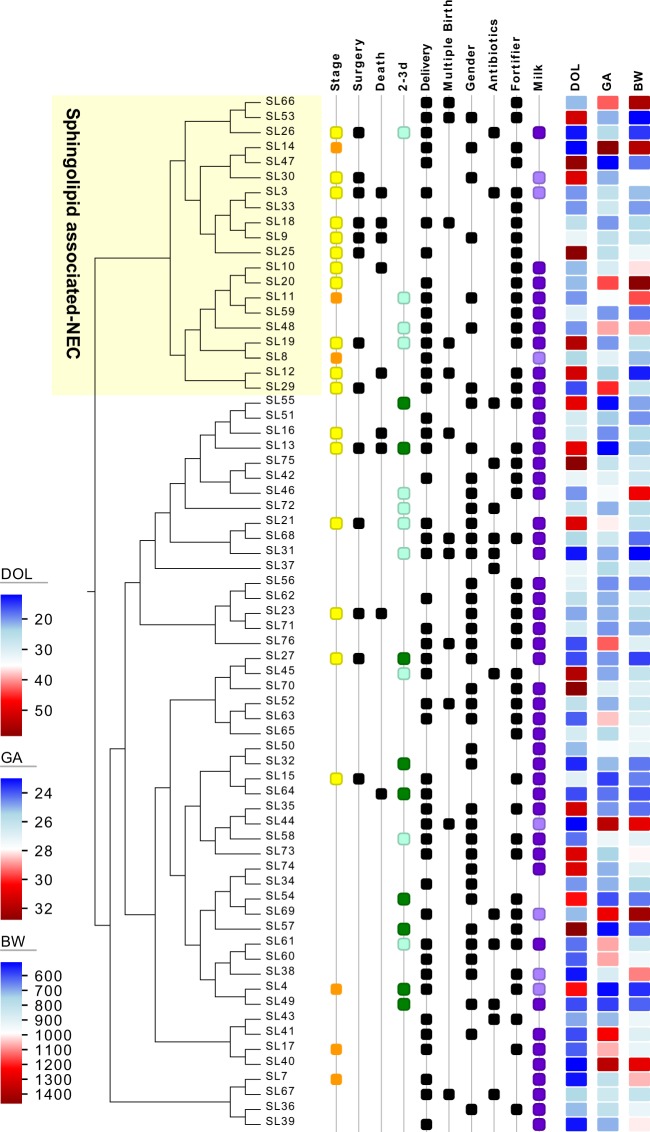
Hierarchical clustering of pre-NEC and control samples. Detected sphingolipids were used for Manhattan distance calculation and hierarchically clustered according to average linkage. More than half the cases cluster separately from 86% of the controls. None of the tested clinical and sample metadata associated with the sphingolipid associated NEC cluster (SLA). NEC Stage II is in orange and Stage III in yellow. When samples were not available 1 day before onset closest sample was selected (2d green, 3d light green). Delivery corresponds to vaginal delivery and gender to female. Infants with mixed feeding (human milk and formula) are represented in light purple. Matched Gestational age (GA) at birth (+/−1 week), day of life (DOL, = /−1), and birthweight (BW) are represented as heatmaps.

**Figure 4:**
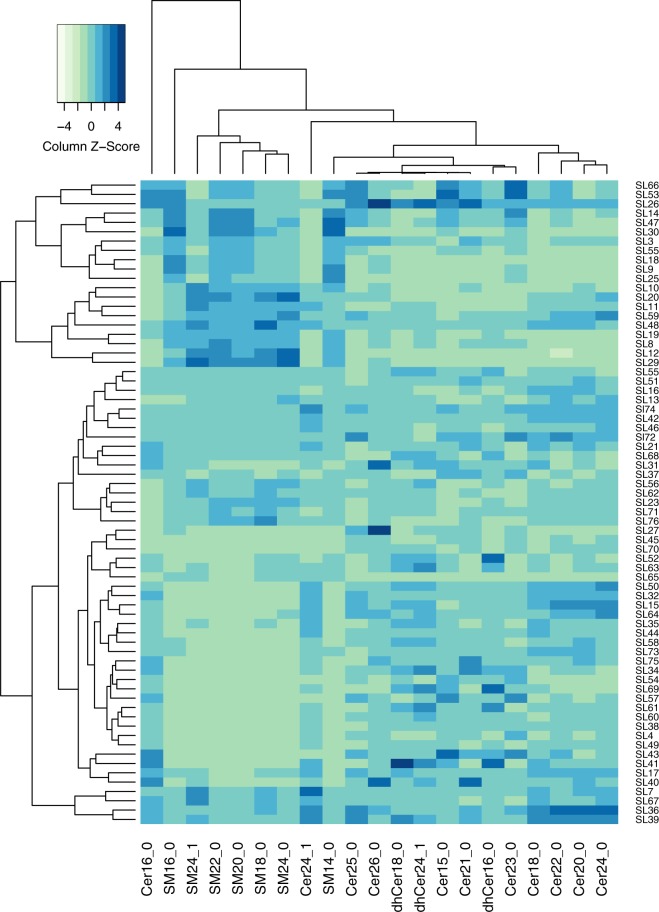
Heatmap of hierarchical clustering of sphingolipids. The sphingolipid-associated NEC cluster is driven by the detected increase in all seven sphingomyelins and decrease in ceramides, especially Cer16:0, 24:1,18:0, 20:0, 22:0, 24:0. Manhattan distance hierarchical clustering with average linkage of scaled metabolites was visualized in a heatmap generated in R.

Because the mechanisms underlying NEC development remain poorly understood, and given the wide range detected for some of the sphingolipids, we decided to pursue the possibility that sphingolipids might play a role in only a subset of the cases. To test this hypothesis, we used hierarchical clustering with average linkage of all the sphingolipids based on Manhattan distance and identified a large cluster of 14 cases with six controls (Figs 3 and 4). The sphingolipid associated (SLA) cluster was retained when including the additional controls and NEC Stage I cases (see Supplementary Fig. S4). Only one case (SL26) and its corresponding controls were lost from the SLA-cluster after adding the ten additional samples. As noted above, the NEC Stage I samples had profiles resembling those of samples from controls (see Supplementary Fig. S4).

**Figure 5:**
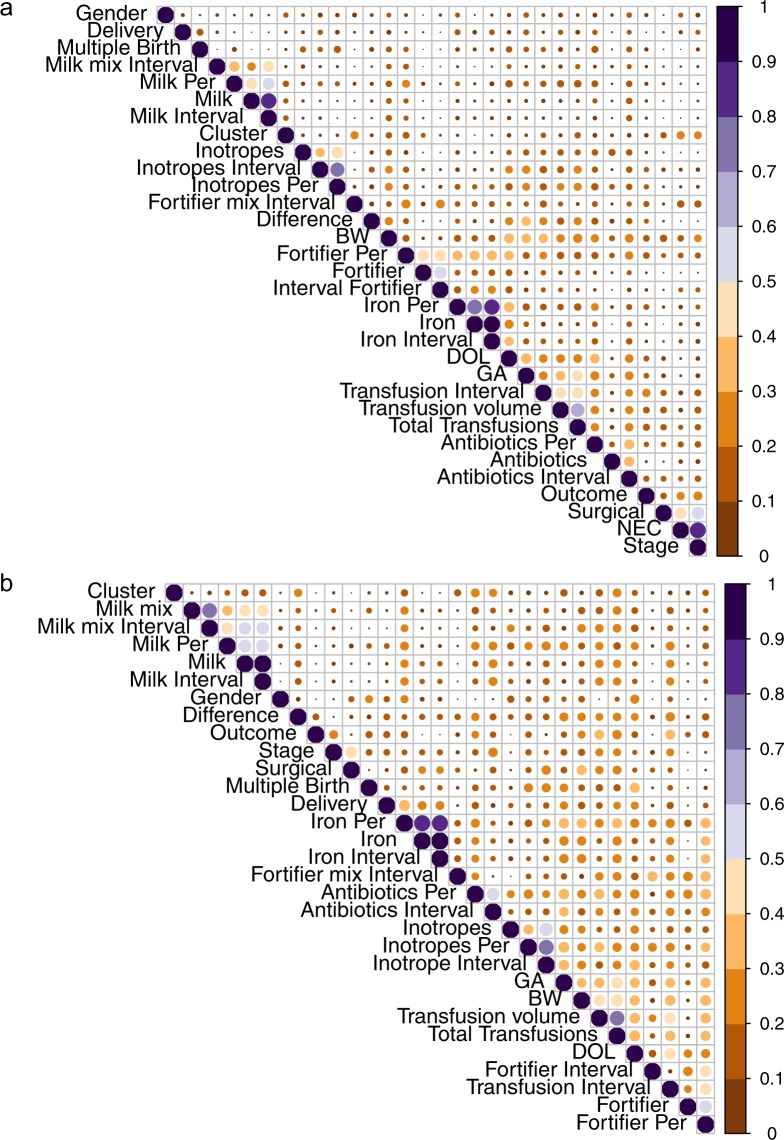
Heatmap of similarity for metadata generated by HAllA. (**a**) No variables had similarities scores >0.5 with the SLA-cluster. Other variables that are associated (case status, Stage of NEC and Surgery) are as expected, as each relates to the disease status; Birthweight, gestational age (GA) at birth, and day of life (DOL) sample was obtained created a cluster as they were fixed for during control selection. All other associations are from temporally stratified feeding and medication information. (**b**) Within cases alone no given variable tested associated with the SLA-cluster. Similarity scores for metadata are visualized in a heatmap generated with corrplots in R. Size and color of circles represent similarity score values.

The SLA-cluster did not include all NEC cases, so we wanted to confirm that the clustering was not associated with other clinical or sample variables. To address that concern, we performed a hierarchical all-against-all association (HAllA) of clinical and sample variables (Table 1) with the SLA-cluster status^43^. The SLA-cluster did not associate significantly with any other variables (Fig. 5a). However, significant associations were found between NEC related variables (Stage, Surgery required), while all other clusters had similarity scores below 0.5 (Fig. 5a, see Supplementary Table S4). As the SLA-cluster consists mainly of cases, we sought to control for possible variable associations that might only be seen within the pre-disease state. When HAllA was run with only the NEC cases, we observed no associations with the hierarchical clustering pattern, but there were significant associations with a similarity score above 0.5 for the temporally stratified variables (Fig. 5b, see Supplementary Table S5). We would like to note that 10 of the 13 cases who underwent surgery had histological confirmation of NEC (in three infants the laparotomy did not yield tissue as the surgeon believed that the extent of the injury was incompatible with survival), but there was no significant enrichment of surgical cases in the SLA-cluster. Because HAllA attempts to group variables that are related to each other, it can identify combinations of variables that associate with the cluster. We therefore consider that the sphingolipid-driven clustering of most of the cases points towards a role of sphingolipids in the development of NEC that cannot be attributed to changes in clinical or sample variables available to us. The hierarchical clustering profile (SLA-cluster, vs remaining samples) was used for classification by machine learning, as previously performed with NEC phenotype. The accuracy of all models was much greater (90–96%) and with smaller standard deviation than for NEC classification except SVMradial (76%) (Table 3). In addition, the false negative and false positive rates were <10% for most models (see Supplementary Table S6).

**Table 3:** Ranking of Machine Learning Models for SLA-Cluster Classification.

Rank							
Iteration	1	2	3	4	5	6	7
1	PLS	RF	KNN	JRip	J48	NB	SVMradial
2	PLS	KNN	RF	JRip	NB	J48	SVMradial
3	PLS	RF	KNN	JRip	J48	NB	SVMradial
4	PLS	KNN	RF	JRip	J48	NB	SVMradial
5	PLS	RF	KNN	JRip	J48	NB	SVMradial
6	PLS	RF	KNN	JRip	NB	J48	SVMradial
7	PLS	KNN	RF	JRip	NB	J48	SVMradial
8	PLS	RF	KNN	NB	JRip	J48	SVMradial
9	PLS	RF	JRip	KNN	J48	NB	SVMradial
10	PLS	KNN	RF	JRip	J48	NB	SVMradial
Total	PLS	RF	KNN	JRip	J48	NB	SVMradial
Average Accuracy	96 ± 8	93 ± 9	93 ± 9	92 ± 10	90 ± 11	90 ± 11	76 ± 16
Average ROC	99 ± 4	99 ± 3	98 ± 6	93 ± 10	92 ± 10	97 ± 7	98 ± 5

KNN: k-nearest neighbor, PLS: partial least squares, RF: random forest, NB: naïve Bayes, SVMradial: support vector machine radial, J48: C4.5-like decision algorithm, JRip: repeated incremental pruning algorithm, ROC: receiver operating characteristic.

## Discussion

Multiple studies have attempted to identify biomarkers for NEC (reviewed in^44^), but no target has advanced to clinical practice. However, timing of the production of the samples analyzed has been problematic. Some biomarker studies used samples collected at NEC diagnosis in attempts to differentiate the clinical deterioration of NEC from that caused by septicemia^45–47^, while other data are derived from specimens obtained up to several weeks before the event^48^. NEC generally occurs within a two-month interval of life, and >90% of very preterm infants do not develop NEC. Hence, considerable infrastructure is required to accrue large numbers of stool samples in anticipation of NEC, but the reality is that only a small subset of infants will experience this outcome, and that this outcome could occur over many weeks. Also, few studies incorporated host variables other than gestational age at birth and age of sample collection.

Our study’s size, and the ability to compare stools from children who soon thereafter developed NEC to stools from well-matched controls, enabled us to examine multiple confounding factors that could influence biomarker association with NEC. We were also able to choose specimens from a highly instructive interval obtained only several days prior to the event. These attributes partially overcame the limitations of prior studies and increased confidence that the perturbed gut sphingolipid metabolism in the interval preceding NEC, compared to matched controls, is relevant to NEC pathogenesis.

Sphingolipids are important components of the cell membrane. They regulate intestinal tight junction integrity and could mediate injurious host response to this dysbiosis^34^. Disrupting the mucosal barrier could increase bacterial translocation and subsequent tissue injury. Altered sphingolipid metabolism increases intestinal permeability and exacerbates experimental colitis^34,49^. In addition, sphingosine-1-phosphate is an important mediator of lymphocyte trafficking and regulates tissue injury in inflammatory bowel diseases^50–56^. Mouse models with different mutations of key enzymes of the sphingolipid pathway demonstrate their harmful^49,53,57,58^ and protective^59^ effects in colitis and colon tumor development. Moreover, compared to other gut metabolites, the source of sphingolipids can be mainly attributed to the host, as only few bacterial taxa produce sphingolipids^60^. Multiple modulators and bioactive sphingolipids are currently in clinical trials for gastrointestinal diseases^61,62^ and might hold value for preventing NEC. The presence of higher levels of sphingolipids might also be related to their reported bactericidal activity against multiple Gram-negative and Gram-positive bacteria^63^ and indirectly by affecting NOD2 recruitment and subsequent innate immunity response^64^.

However, despite this large sample size and strategically focused interval of analysis, i.e., 1–3 days before NEC developed for the targeted assays, we encountered considerable data dispersion around the median values in the control samples. This hindered our ability to generate a machine learning classification model with sufficient accuracy to apply to at-risk infants. We cannot attribute the wide range of sphingolipid concentrations (see Supplementary Fig. S2) to a subset of the controls having unrecognized NEC I disease, because specimens from infants that were designated as having NEC I had sphingolipid and ceramide profiles that resembled the profiles from the control specimens. This finding, however, suggests that NEC Stage I is probably not on the same continuum with NEC Stages II and III^41,42^.

Interestingly, more than half of the cases clustered into a subgroup that we could not attribute to any other clinical variable. This cluster contained only a limited number of controls and performed very well in machine learning. Sphingolipids could still be valuable for monitoring of at least a subset of NEC cases, as the sphingolipid-based clustering is highly enriched in pre-event samples. Nevertheless, our data suggest that a combination of sphingolipids with other clinical or possibly metabolic variables should be pursued to better capture the at-risk population at large.

We considered that the differences in sphingolipids could be related to dietary differences between cases and controls, but we found no association between NEC and feeding variables, such as presence of human milk or fortifier. Hence, this strengthens the case for an endogenous over a dietary source of the altered gut sphingolipids. We also recognize that dietary sphingolipids might be relevant to NEC development as they can alter gut microbiome composition^65,66^. Additionally, gangliosides (glycosphingolipids) in colostrum protect against intestinal injury in animal models^67,68^. Colostrum was systematically used for initial feedings, although not separately recorded from human milk. However, only three cases and corresponding controls were collected before the first two week of life, a time when most available maternal colostrum is depleted. None of the early time point (<2 weeks) controls and only two of the cases fell within the SLA-cluster (Fig. 3). Therefore, colostrum is unlikely to have contributed to findings, but we cannot unequivocally exclude this possibility, either.

We wish to acknowledge some limitations to our data. First, an analysis of the St. Louis cohort^22^ identified a dysbiotic state consisting of over-represented Gammaproteobacteria and under-represented Negativicutes prior to NEC onset. The analysis did not identify bacteria capable of producing sphingolipids. However, a direct comparison of the microbial community in samples obtained just before onset was not performed and therefore we cannot completely exclude contributions by the microbiota to the fecal sphingolipid pool. We plan to address this possibility by performing extended 16 S and metagenomic analysis to confirm that there is minimal or no potential to synthesize sphingolipids in the microbial population. Bacterial sphingolipids are highly relevant to the developing gut as they modulate invariant natural killer T cell activation and presence in the gut^37,69^. Simultaneously, we will attempt to determine if altered sphingolipid levels have effects on the microbial community. Imbalances in sphingolipid metabolism could drive the dysbiotic state observed prior to onset, either by directly affecting the bacteria or through their signaling function in the immune response. Another potential limitation is that we focused on stool. While it seems logical to seek biomarkers in specimens that have just passed through the organ of interest, i.e., the infant gut, the possibility exists that blood or urine are more appropriate substances for such analyses. However, the difficulties of acquiring blood or urine from preterm infants compared to stool limits their applicability to biomarker studies in a prospective context. Finally, we made a strategic decision to study materials obtained several days before the event, postulating that this proximal pre-NEC interval would have the greatest yield in a search for biomarkers. Though it seems unlikely, we cannot exclude the possibility that informative but ephemeral biomarkers are present well before then, i.e., hit-and-run indicators of subsequent development of NEC.

Most current analyses of complex biologics such as stools rely on nucleic acid sequencing, and the readouts of such analyses have limitations. Specifically, microbial DNA sequences can only infer the metabolic capacity of a polymicrobial substance, and microbial and host mRNA sequences reflect transcriptional physiology but do not account for subsequent gene product modification or activation. Our work exemplifies the opportunities of applying broad range metabolomics technology to such analytes. Most notably, by identifying molecules associated with sphingolipid pathways, one gains confidence that the process actually plays a role in the phenotype of interest. However, metabolomics analyses pose challenges, as broad range screens are not quantitative, per-specimen cost is high, and the resulting data require considerable computational interrogation to detect associations. Moreover, validation of presence of the pathway suggested by broad range metabolomics and scale-up to larger specimen sets depend on assays that are specific to the molecules of interest, and which are often more complex than PCR quantification of mRNA abundance.

In summary, our data suggest that sphingolipid metabolism is worthy of pursuit in animal models of early life gut injury that resemble NEC. However, in view of the many challenges of obtaining and analyzing specimens with predictive value for subsequent development of NEC, biomarker discovery is likely to remain an elusive goal.

## Methods

### Participants

The specimens used in this study were acquired from a prospective cohort study at St. Louis Children’s Hospital (SLCH). Before specimen acquisition and enrollment, a written informed consent was obtained from parents. The study was approved by the Washington University Human Research Protection Office. Specimen management and case definition have been described previously^22,25,70^. Briefly, only preterm infants with birthweights ≤1500 g hospitalized at the SLCH neonatal intensive-care unit were enrolled after obtaining written informed consent from their parents. Stool was collected until discharge. Cases were defined as infants with Bell’s Stage II or III NEC, who had no congenital heart defects or spontaneous intestinal perforation, unless there was subsequent radiographic confirmation of necrotizing enterocolitis. In this study, we used study participants from Warner *et al*.^22^ (see Supplementary Fig. S1, Supplementary Table S1). Controls were matched by gestational age at birth (+/−1 week), birthweight (+/−100 g) and day of life (+/−1 day). Controls with bacterial bloodstream infections, congenital heart defects or spontaneous intestinal perforation were excluded.

During study design for some of the participants repeated samples were selected to determine temporal changes in metabolites. From this cohort we included in the broad range metabolomics analysis the specimen from each case that was closest to the onset of NEC (one sample per case), limited to the one to five-day window before NEC developed (see Supplementary Table S1). We did not analyze stools obtained on the day NEC occurred, because we wanted to avoid specimens from infants in which NEC was already evolving. From the matched controls, we analyzed stools produced on, or, if necessary, within five days before, the day of life on which NEC occurred in the corresponding case (see Supplementary Table S1). For the follow-up targeted metabolomics, we expanded the set to a total of 23 pre-event stools matched to 46 additional control stools (see Supplementary Fig. S1, Supplementary Table S1). Pre-NEC stools were selected according to availability, but within a window of one to three days prior to the event. Controls were matched as for the broad range metabolomics analysis at the same day of life as the case (one sample per case and control study participant), except for one sample due to limited stool availability. In all situations, we did not use samples where the remaining stool was <2.5 g. Also, in no case did we sample on the day NEC was diagnosed.

### Broad Range Metabolomics

Metabolon (Durham, NC) performed broad range metabolomics as published^71^. A detailed description of the extraction and processing is provided in supplementary material. Briefly, fractions from pre-event stools samples were subjected to four different ultra-performance liquid chromatography-tandem mass spectrometry (UPLC-MS/MS) conditions to detect acidic, basic, and hydrophobic compounds in both positive and negative modes. The detected compounds were compared to the proprietary metabolite database containing more than 30,000 curated entries. Identified metabolites were reported as area-under-the-curve and used for statistical analyses.

### Analysis of Broad Range Metabolomics

Statistical interrogations were performed in R^72^. Metabolites with >20% missing values were excluded from analysis. Peak area ratios were subjected to probabilistic quantile normalization (PQN) according to Di Guida *et al*. for univariate analysis^73^. Normalized data were compared by a two-sided Welch’s test with false discovery rate correction and fold changes. A metabolic network was built in MetaMapR based on KEGG and PubChemID information^74^. Edges were imported in Cytoscape and visualized with p-values and fold change^75^. To identify pathways that were enriched in changes between cases and controls we adapted the GSEA-PCA method used by Morgan *et al*. to metabolites^76^. The GSEA matrix was replaced with a metabolite matrix based on KEGG modules relationships. After removing metabolites with >20% missing values and PQN normalization, remaining missing values were imputed with random forest. Metabolites with variances in the two lowest quantiles were removed. We performed a PCA of the metabolites that included underlying pathway information. Components with at least one metabolite in the pathway passing the Benjamini Hochberg (BH) corrected p-value were visualized in a heat-map.

### Targeted Metabolomics

A 10 mg stool aliquot was suspended in 2% CHAPS (Sigma-Aldrich). Ceramide C17 (Avanti Lipids) (20 μg/sample) and sphingomyelin 18:1-d9 (Avanti Lipids) (0.2 μg/sample) in methanol were added as internal standards. We precipitated proteins by vigorously shaking the suspension (3 min) followed by centrifugation (10 min, 9,000 g). The supernatant was phase extracted by the Bligh-Dyer technique^77^ using methanol:chloroform:0.5 M NaCl (1:1:1) and vigorous shaking (3 min), followed by centrifugation (10 min, 620 g at 4 °C). The chloroform phase was aspirated, desiccated with N_2_ and resuspended in methanol:chloroform (9:1). A fraction of each sample was pooled for quality control (QC). The QC pool was injected six times in the beginning of a run to stabilize the instrument, and again once between every ten study samples. Sphingolipids with a CV < 15% in the QC sample were reported. Sphingolipids were measured with a Shimadzu 10 A HPLC system and a Shimadzu SIL-20AC HT auto-sampler coupled to a Thermo Scientific TSQ Quantum Ultra triple quadrupole mass spectrometer operated in SRM mode under ESI(+). Data processing was conducted with Xcalibur (Thermo Fisher). The relative quantity of each detected sphingolipid was reported as a peak area ratio.

### Statistical Analysis and Machine Learning of Sphingolipids

Peak area ratios for each sphingolipid were scaled in R for improved visualization^72^. Statistical significance by two-sided Welch’s t-test corrected for multiple comparison with BH method was determined in R. Data were visualized with GraphPad Prism 7 (GraphPad Software). Unscaled data were used for hierarchical clustering based on Manhattan distance with average linkage in the specmine package^78^. Hierarchical clustering was visualized with metadata in Evolview^79,80^ and as heatmap with the gplots package v. 3.0.1^81^. Resulting clusters were used for classification in machine learning and compared to NEC status-based classification. Required normalization was performed by autoscaling the sphingolipid levels. The following machine learning algorithms were tested: PLS, J48, JRip, SVMradial, RF, NB, KNN. We modified the training functions in the specmine package in R to account for downsampling and used 10 fold cross-validation^78^. The analysis was repeated 10 times with set seeds to determine reproducibility. We used accuracy and confusion matrices to rank the tested algorithms, and Hierarchical All-against-All association testing (HAllA)^43^ to determine associations among clinical variables for cases only or the complete dataset. Similarity values were visualized with a modified corrplot in R^82^. Table 1 provides an overview of the clinical and sample variables applied in HAllA. Dimensions were reduced by medoid decomposition (see Supplementary Table S7) and analyzed by normalized mutual information. Targeted metabolomics data are available from the corresponding author on reasonable request.

### Disclosures

P.I.T. MediBeacon (consultant, Scientific Advisory Board, equity), co-inventor of patent relevant to transcutaneous monitoring of fluorophores to measure gut permeability and potential recipient of royalty payments from this technology. All other authors have no disclosures.

## Electronic supplementary material

Supplementary Materials

S1 Dataset

S2 Dataset

S7 Dataset
