# Supplementary material for: Gut Sphingolipid Composition as a Prelude to Necrotizing Enterocolitis
Source: Sci Rep. 2018 Jul 20;8:10984. doi: 10.1038/s41598-018-28862-4 (PMC6054655; doi:10.1038/s41598-018-28862-4)
Supplement: Supplementary file 1 — Supplementary Materials [file 41598_2018_28862_MOESM1_ESM.pdf]

Supplementary Information: Gut Sphingolipid Composition as a Prelude to Necrotizing Enterocolitis

B. Rusconi[1], X. Jiang[2], R. Sidhu[2], D.S. Ory[2], B.B. Warner[1], P.I. Tarr[1,3]
[1] Department of Pediatrics, [2] Department of Medicine, [3]Department of Molecular Microbiology, Washington University in St. Louis School of Medicine, St. Louis, MO USA

Correspondence to:

Phillip I. Tarr, Department of Pediatrics, Washington University in St Louis School of Medicine, St Louis, MO 63110, USA tarr@wustl.edu

Supplementary Material

Broad Range Metabolomics

A recovery standard was added to samples extracted with the automated MicroLab STAR® system. Protein precipitation was performed with methanol under vigorous shaking for 2 min and subsequent centrifugation. Supernatant was separated into five fractions; one for backup and four for the different analysis conditions. All samples were analyzed on a Waters ACQUITY ultra-performance liquid chromatography (UPLC) and a Thermo Scientific Q-Exactive high resolution/accurate mass spectrometer (MS) interfaced with a heated electrospray ionization (HESI-II) source and Orbitrap mass analyzer operated at 35,000 mass resolution. For acidic positive ion conditions the samples were gradient eluted from a C18 column (Waters UPLC BEH C18-2.1x100 mm, 1.7 µm) using water and methanol, containing 0.05% perfluoropentanoic acid (PFPA) and 0.1% formic acid (FA). For hydrophobic compounds the same column was used, but gradient eluted with methanol, acetonitrile, water, 0.05% PFPA and 0.01% FA. Basic compounds were gradient eluted from a separate C18 column using methanol and water with addition of 6.5mM ammonium bicarbonate at pH 8 and analyzed with negative ion conditions. The fourth fraction was eluted from a HILIC column (Waters UPLC BEH Amide 2.1x150 mm, 1.7 µm) using a gradient consisting of water and acetonitrile with 10mM ammonium formate, pH 10.8, and analyzed in negative ion mode. The MS scan range used in all four methods covered 70-1000 m/z. QC samples of pooled samples were acquired frequently throughout the run to verify consistency of acquisition. Peaks of raw data were identified and processed with Metabolon's hardware and software. Compound identification was based on comparison to Metabolon's library of purified standards and recurrent unknown entities. Additional criteria for identification were retention index, accurate mass match to library +/- 0.005 amu, and the MS/MS forward and reverse scores between the acquired data and standards. A data normalization step was performed to correct variation resulting from instrument inter-day tuning differences. Identified metabolites were reported as area-under-the-curve.

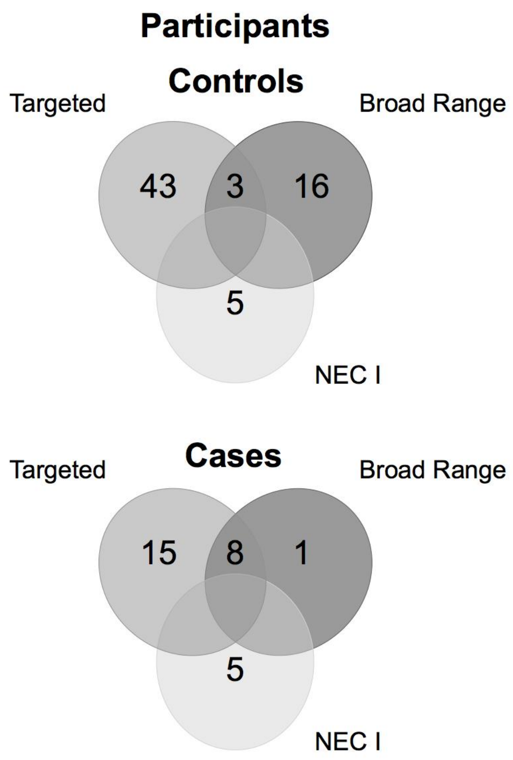

Supplementary Figure S1. Participant and Sample Overview. Overlap between participants selected for broad range metabolomics and targeted MS was limited within the controls. Multiple samples from the same participant were initially processed in the broad range analysis. Although selected cases overlapped between broad range and targeted the processed samples differed after restricting the time interval to 1-3 days before onset and availability.

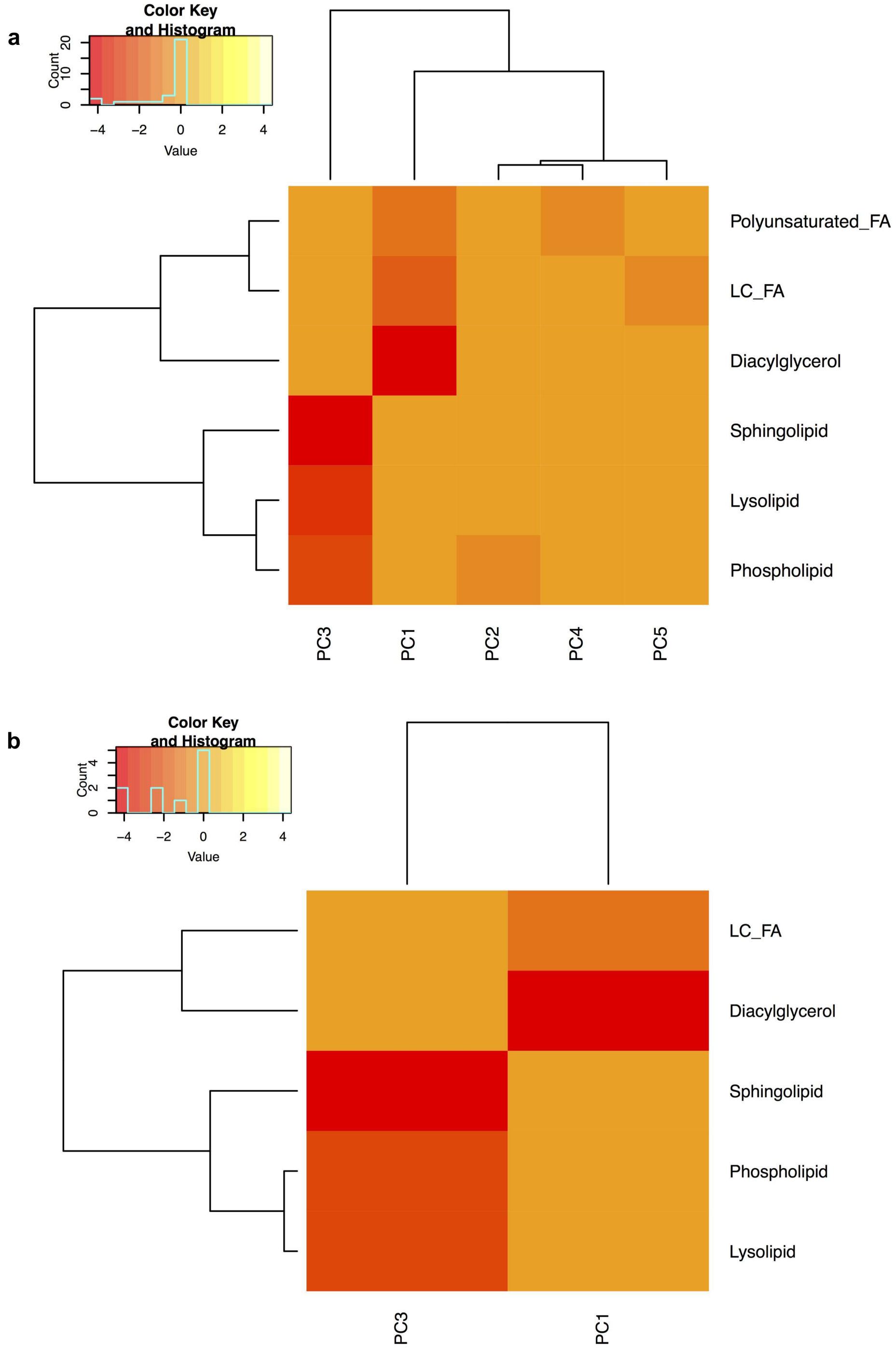

Supplementary Figure S2. Heatmap of FDR or Bonferroni-corrected KEGG pathway weighted PCs of pre-event metabolites. Pathway information was included in PCA of pre-event stool metabolites from cases and controls. Heatmap only displays pathways with at least one significant member ($\log_{10}$ of <0.1) after (**a**) false discovery rate correction (Benjamini Hochberg-method) or (**b**) after Bonferroni correction. All the pathways identified belong to the lipid supergroup, such as long chain fatty acids (LC_FA).

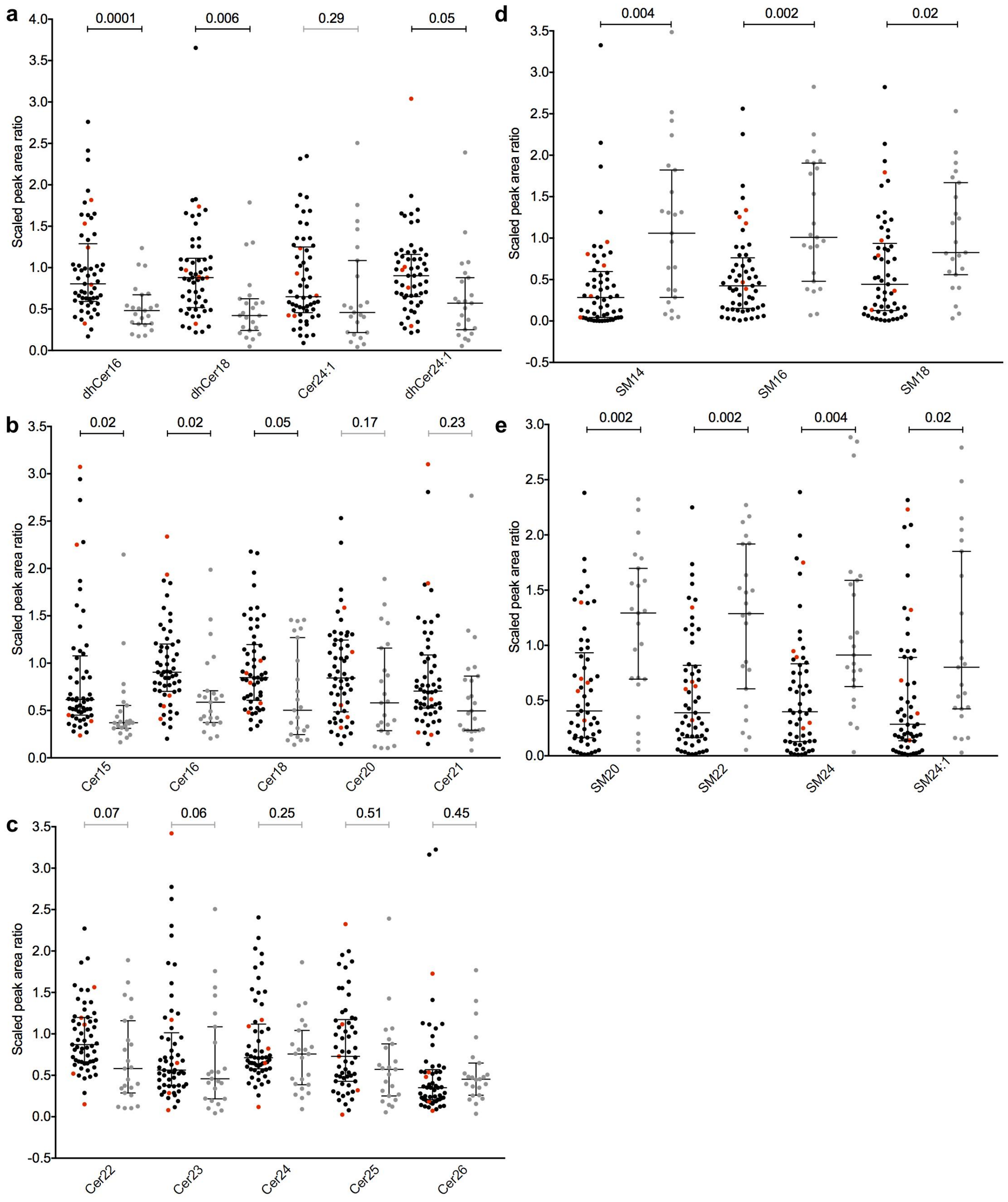

Supplementary Figure S3. Targeted sphingolipid detection in pre-event stool. NEC stage I cases did not fall into the range observed in the cases and were, instead, spread throughout the values for the controls. (**a-c**) Scaled peak area ratios of ceramides to internal standard ceramide (Cer17). (**d, e**) Scaled peak area ratios of sphingomyelins to deuterated internal standard sphingomyelins (SM18:1-d9). Scatterplots of scaled values with median and interquartile range were compared by two-sided Welch's t-test with BH multiple test correction ($p<0.05$). Black circles represent controls, grey circles represent cases, and red circles represent NEC Stage I.

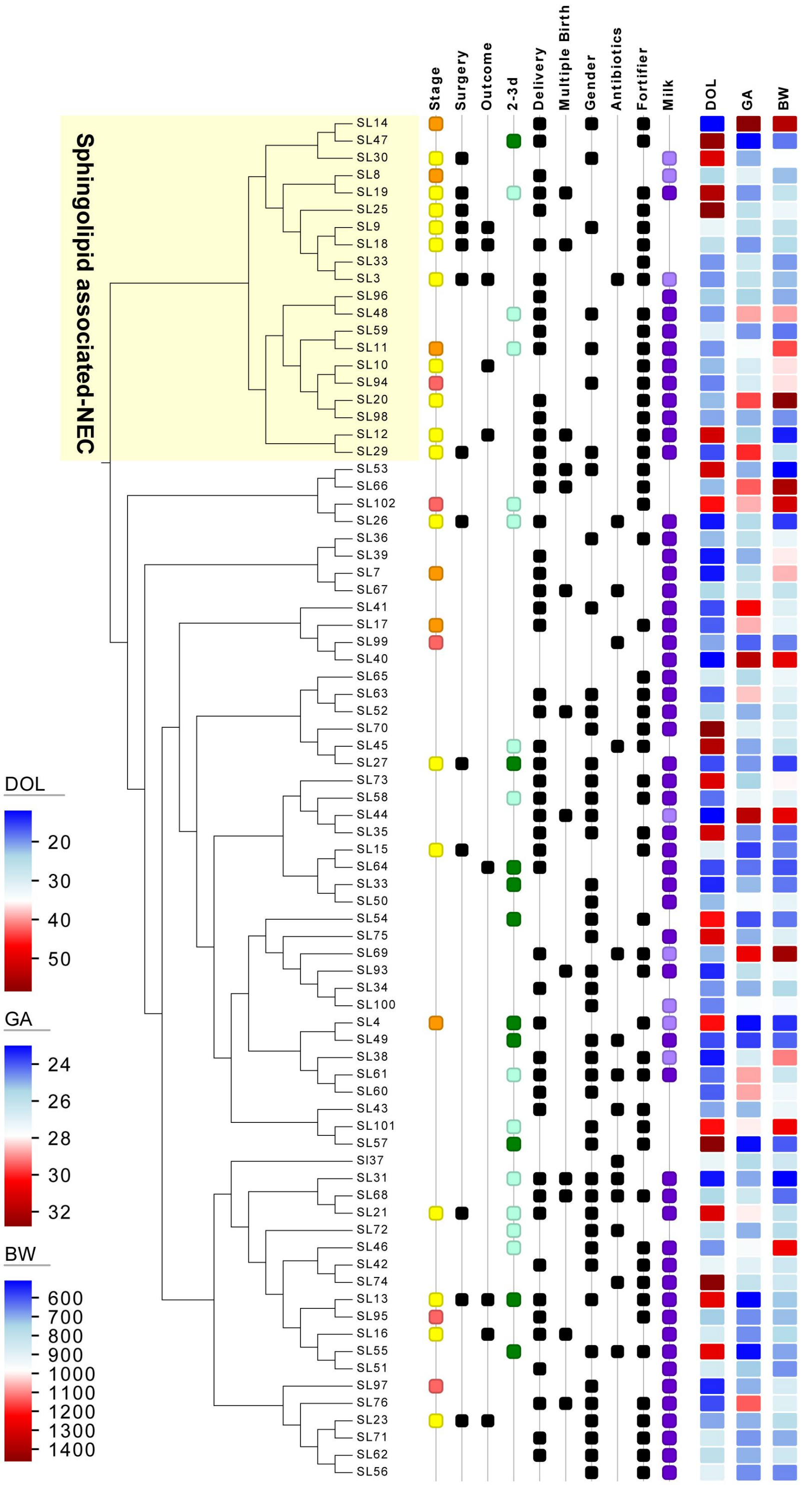

Supplementary Figure S4. Hierarchical clustering of pre-NEC and control samples including NEC Stage I. NEC Stages I, II, and III are in light red, orange, and yellow, respectively. When samples were not available 1 day before onset, we selected the next closest sample (2 day before onset: light green, 3 days before onset green). Delivery corresponds to vaginal delivery and gender to female. Infants with mixed feeding are depicted in light purple.

Table S3. Average False Positive and False Negative Rates for NEC Classification

Models	False Positives	False Negatives
J48	29.8	32.5
nb	28.4	33.3
rf	31.6	33.7
JRip	25.8	35.4
plS	24.2	36.5
knn	18.2	39.5
SVMradial	40.4	47.8

Table S4. Similarity Scores for Clinical and Sample Variables

Similarity score between Clusters	Variables	Similarity Score	Variables	Similarity Score	p-value	q-value
0.55	NEC;Stage	0.88	Surgical	1.00	0	0
0.24	Interval Fortifier mix	1.00	Fortifier %; Fortifier; Interval Fortifier	0.47	0	0
0.23	Outcome	1.00	NEC;Stage	0.88	0	0
0.23	NEC;Stage	0.88	Outcome	1.00	8.26E-11	6.07E-10
0.20	Difference	1.00	BW;DOL;GA	0.31	0.001	0.007
0.20	Cluster	1.00	Interval Fortifier mix	1.00	0	0
0.18	NEC;Stage	0.88	Cluster	1.00	0.0004	0.003
0.15	NEC	1.00	Interval Fortifier mix	1.00	0	0
0.15	Interval Fortifier mix	1.00	NEC;Stage	0.88	0.003	0.02
0.14	BW;DOL; GA	0.31	Fortifier %;Fortifier; Interval Fortifier	0.47	0.002	0.01
0.13	Delivery	1.00	Multiple Birth	1.00	0	0
0.13	Multiple Birth	1.00	Delivery	1.00	0.001	0.007
0.08	Cluster	1.00	Interval HM mix;HM % ;HM;Interval HM	0.38	0.001	0.007

Table S5. Similarity Scores for Clinical and Sample Variables of Cases

Similarity score between Clusters	Variables	Similarity Score	Variables	Similarity Score	p-value	q-value
1	Stage; Surgical	0.45	Stage; Surgical	0.45	0	0
1	HM %; HM; Interval HM	0.54	HM %; HM; Interval HM	0.54	0	0
1	HM mix; Interval HM mix	0.72	HM mix; Interval HM mix	0.72	0	0
1	Interval Fortifier; Fortifier; Fortifier %	0.42	Interval Fortifier; Fortifier; Fortifier %	0.42	0	0
1	Antibiotics %;Interval Antibiotics	0.51	Antibiotics %;Interval Antibiotics	0.51	0	0
0.5	HM %; HM; Interval HM	0.54	HM mix ;Interval HM mix	0.72	0	0
0.5	HM mix; Interval HM mix	0.72	HM %; HM; Interval HM	0.54	0	0

Table S6. Average False Positive and False Negative Rates for SLA-Cluster

Models	False Positives	False Negatives
J48	10.8	5
RF	6.8	5.7
JRip	9.1	5.9
PLS	2.9	6.9
NB	9.5	9.3
KNN	4.2	12.5
SVMradial	19.4	34.2
